# Microinvasive pars plana vitrectomy combined with internal limiting membrane peeling versus anti-VEGF intravitreal injection for treatment-naïve diabetic macular edema (VVV-DME study): study protocol for a randomized controlled trial

**DOI:** 10.1186/s13063-023-07735-w

**Published:** 2023-10-24

**Authors:** Haoxin Guo, Wenbo Li, Zetong Nie, Xiang Zhang, Mingfei Jiao, Siqiong Bai, Naxin Duan, Xiaorong Li, Bojie Hu

**Affiliations:** Tianjin Key Laboratory of Retinal Functions and Diseases, Tianjin Branch of National Clinical Research Center for Ocular Disease, Eye Institute and School of Optometry, Tianjin Medical University Eye Hospital, Tianjin, 300384 China

**Keywords:** Diabetic macular edema, Vitrectomy, Anti-VEGF, Study protocol, Randomized controlled trial

## Abstract

**Background:**

Diabetic macular edema (DME) is the main cause of vision loss in diabetic patients. Currently, anti-vascular endothelial growth factor (VEGF) intravitreal injection stands as the first-line therapy for DME. However, some patients exhibit insufficient response to anti-VEGF agents and often require multiple injections, imposing psychological and economic burdens. While microinvasive pars plana vitrectomy (PPV) has been shown to be safe and effective in treating refractory DME, scant research has explored its application to treatment-naïve DME. The purpose of this study is to determine whether early PPV combined with internal limiting membrane (ILM) peeling can lessen the therapeutic burden of DME patients, prevent vision loss, and maintain long-term stabilization of diabetic retinopathy.

**Methods:**

This is a single-center, prospective, parallel-group, non-inferiority randomized controlled trial involving 102 DME participants. Participants will be randomly assigned to either the study group (PPV combined with ILM peeling) or the control group (conbercept intravitreal injection (IVC)) at a 1:1 ratio, with a scheduled follow-up at 12 months post-operation. Comparative analysis of results between the two groups will be conducted at months 1, 3, 6, and 12 after the intervention. The primary outcomes involve evaluating the changes in central subfield thickness (CST) and best corrected visual acuity (BCVA). The secondary outcomes include assessment of optical coherence tomography (OCT) and OCT angiography (OCTA) biomarkers, re-treatment and adverse events rates, diabetic retinopathy (DR) development, cost-effectiveness analysis, and vision-related quality of life (VRQL).

**Discussion:**

Some patients do not respond well to anti-VEGF drugs and repeated intravitreal injections increase the treatment burden for patients. The VVV study aims to explore whether PPV combined with ILM peeling could become an initial treatment option for treatment-naïve DME patients.

**Trial registration:**

ClinicalTrials.gov NCT05728476. Registered on 15 February 2023.

## Administrative information

Note: the numbers in curly brackets in this protocol refer to SPIRIT checklist item numbers. The order of the items has been modified to group similar items (see http://www.equator-network.org/reporting-guidelines/spirit-2013-statement-defining-standard-protocol-items-for-clinical-trials/).

**Table:** 

Title {1}	Microinvasive pars plana vitrectomy combined with internal limiting membrane peeling versus anti-VEGF intravitreal injection for treatment-naïve diabetic macular edema (VVV-DME study): study protocol for a randomized controlled trial
Trial registration {2a and 2b}.	Clinical.Trials.gov identifier: NCT05728476, registered on 15 February 2023, https://classic.clinicaltrials.gov/ct2/show/NCT05728476
Protocol version {3}	Version 2.0, 4 October 2023
Funding {4}	Funded by Tianjin Key Medical Discipline (Specialty) Construction Project (No.TJYXZDXK-037A), Natural Science Foundation of Tianjin City (No.20JCZXJC00040), and Science&Technology Development Fund of Tianjin Education Commission for Higher Education (No.2022ZD058).
Author details {5a}	Haoxin Guo^1^, Wenbo Li^1^, Zetong Nie^1^, Xiang Zhang^1^, Mingfei Jiao^1^, Siqiong Bai^1^, Naxin Duan^1^, Xiaorong Li^1^, and Bojie Hu^1^ 1 Tianjin Key Laboratory of Retinal Functions and Diseases, Tianjin Branch of National Clinical Research Center for Ocular Disease, Eye Institute and School of Optometry, Tianjin Medical University Eye Hospital, Tianjin 300384, China Correspondence: Professor Bojie Hu; bhu07@tmu.edu.cn; http://orcid.org/0000-0001-7840-8290
Name and contact information for the trial sponsor {5b}	Investigator initiated clinical trial Bojie Hu, Email: bhu07@tmu.edu.cn
Role of sponsor {5c}	This is an investigator initiated clinical trial. The funders had no part in the design, data collection, analysis, interpretation, or writing of the manuscript.

## Introduction

### Background and rationale {6a}

Diabetic macular edema (DME), characterized by the accumulation of exudative fluid in the macula, is the leading cause of vision loss in diabetic patients [1]. With optical coherence tomography (OCT), approximately 5.47% of diabetic patients can be diagnosed with DME [2]. Given the rising prevalence of diabetes [3], DME is becoming an increasingly significant public health concern.

Although the pathogenesis of DME remains unclear, over-expression of vascular endothelial growth factor (VEGF) and over-release of inflammatory cytokines are associated with DME according to recent studies [4]. Focal/grid laser treatment was formerly the gold standard for treating DME, but it could only reduce the chance of moderate vision loss and most patients could not regain lost vision after undergoing this procedure [5]. In contrast, vitreous injection of anti-VEGF has demonstrated greater effectiveness and has become the first-line therapy in treating DME [6]. Conbercept is a 141-kDa engineered fusion protein that has a high affinity for binding all isoforms of VEGF-A, VEGF-B, VEGF-C, and PIGF [7]. Single conbercept intravitreal injection (IVC) can reduce ocular VEGF concentration for more than 60 days [8]. Previous studies have shown the effectiveness and safety of IVC in DME treatment [9–11]. However, a considerable proportion of patients do not react effectively to anti-VEGF agents and often require multiple injections [12]. Refractory or persistent DME refers to eyes that have undergone 4 anti-VEGF injections within 6 months and have a persistent central subfield thickness (CST) of 250 μm or more on OCT scans, as determined by the Diabetic Retinopathy Clinical Research Network (DRCR.net) [13]. After 2 years of monthly injections, over 40% of patients in DRCR protocol I had refractory DME [14], and at least 26% patients in RISE/RIDE study had the same issue [15]. Obviously, refractory DME is not uncommon and typically requires additional treatment, elevating the psychological and economic burden of patients [16]. In addition, repeated injections may lead to degeneration of the remaining healthy retinal nerves, increasing the risk of choroidal capillary circulatory disturbance [17]. As a result, there is a great demand for alternative therapy that can effectively manage DME with lower treatment burden.

Lewis et al. firstly reported pars plana vitrectomy (PPV) as a therapy for tractional DME in 1992 [18]. Microinvasive vitreous surgery and an optimized vitrectomy platform with fluid and pressure control have greatly reduced the complications of PPV, resulting in a relatively low complication rate of approximately 1%, which is similar to the cumulative and serious complication rate associated with repeated anti-VEGF injections [19, 20]. The possible mechanisms of PPV for DME include relieving the mechanical traction of macula, increasing the oxygenation within the vitreous cavity, and promoting the clearance of VEGF [21–25]. PPV is currently proposed when traditional therapies fail in treating refractory DME cases that are accompanied by posterior vitreous cortical retraction, hard exudates in the fundus, and vitreous macular traction [26–28]. When combined with ILM peeling, PPV can release mechanical traction, completely remove the residual posterior vitreous cortex that cannot be cleared by traditional PPV, and prevent the formation of epiretinal membrane [29]. In addition, PPV combined with ILM peeling can reduce the attachment of vasoactive and inflammatory factors, leading to a lower incidence of vitreoretinal interface disease postoperatively [30–33]. Studies have shown that PPV combined with ILM peeling is safe and efficient for both tractional and non-tractional DME [30, 34–36]. However, in previous studies, PPV was mostly considered to be a last resort for DME. Due to failed treatments and a long course of the disease, the photoreceptors as well as the outer membrane have been damaged, thus limiting the therapeutic effect [37–39].

OCT and OCT angiography (OCTA) have become essential techniques in identifying biomarkers for DME, which have been demonstrated to be closely correlated with therapeutic efficacy. Many studies have demonstrated the correlation between various imaging biomarkers and the response to anti-VEGF drug treatment, including subretinal fluid (SRF), location and size of cystoid change, continuity of the inner segment-outer segment (IS-OS) layer, hyperreflective foci (HRF), and the status of the vitreomacular interface in OCT [40–42], as well as the foveal avascular zone (FAZ) and retinal blood flow density in OCTA [43–45]. Nevertheless, there exists a paucity of examining imaging biomarkers in treatment-naïve DME patients undergoing vitrectomy. And only the VITAL study has identified the presence of SRF as a predictive anatomical factor for better visual outcomes [36]. Therefore, our study aims to further explore which biomarkers are more suitable for surgical treatment compared to anti-VEGF drug treatment.

### Objectives {7}

#### Main objective

The main objective is to compare the changes in best corrected visual acuity (BCVA) and CST in patients with treatment- naïve DME who undergo PPV combined with ILM peeling versus those who receive conbercept intravitreal injection.

#### Secondary objectives

The secondary objectives include assessment of OCT and OCTA biomarkers, diabetic retinopathy (DR) development, re-treatment, adverse events, cost-effectiveness analysis, and vision-related quality of life (VRQL) of the two groups.

### Trial design {8}

**Fig. 1:**
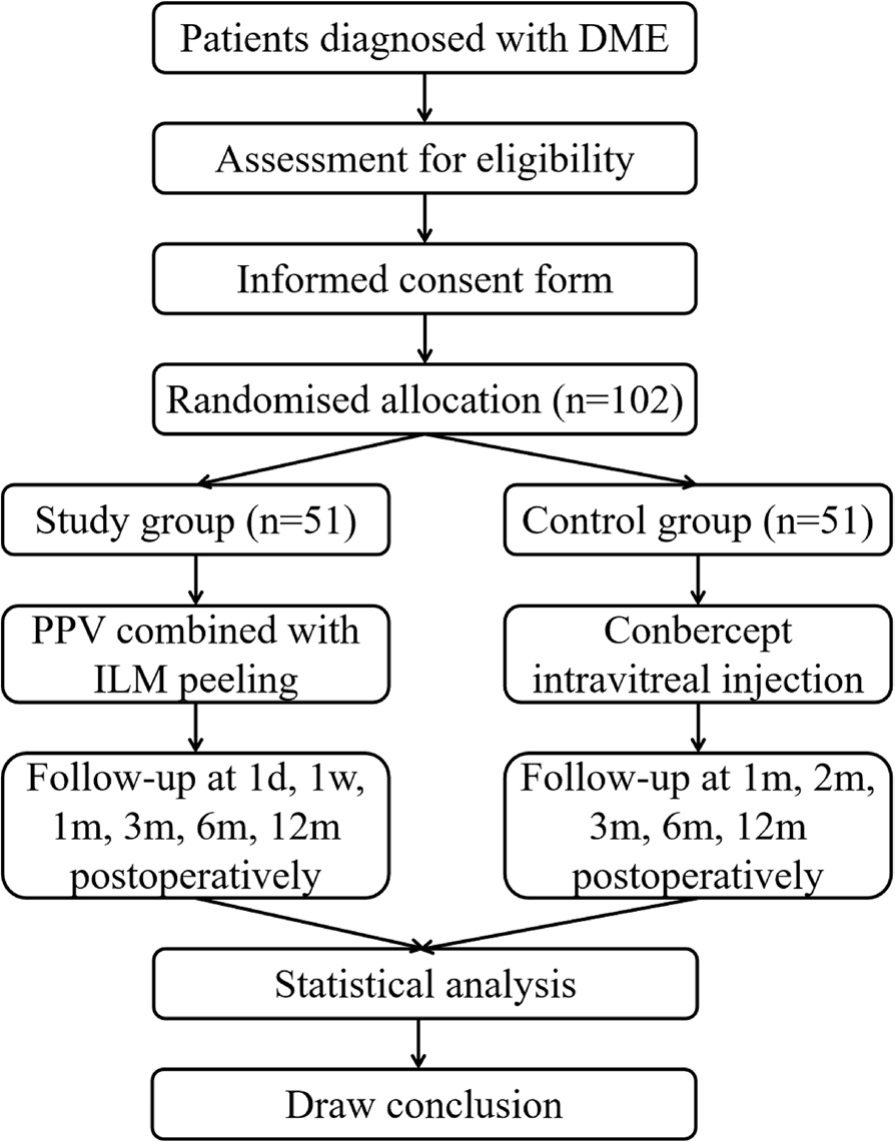
Trial design flow chart. DME, diabetic macular edema; PPV, pars plana vitrectomy; ILM, internal limiting membrane; d, day; w, week; m, month

VVV is a single-center, prospective, parallel-group, non-inferiority, randomized controlled trial. Enrolled patients will be randomly assigned at a 1:1 ratio into the study group (PPV combined with ILM peeling) and control group (IVC), and a follow-up period of 12 months is planned. Interim analyses will not be conducted. Figure 1 depicts the trial design flow chart.

## Methods: participants, interventions, and outcomes

### Study setting {9}

This study will be carried out during November 1, 2023, to November 1, 2025, at Tianjin Medical University Eye Hospital in Tianjin, China. The hospital is a class A tertiary hospital specializing in the treatment of eye diseases and provides services to patients from Tianjin and the surrounding areas. The recruitment, surgery, and follow-up will all take place here. A dedicated investigator will communicate with patients who are eligible for our inclusion about the specifics of the trial. Before undergoing any interventions related to this research, each participant must sign an informed consent form. Researchers will present the trail in popular expression to ensure that participants can fully understand the benefits as well as potential harms and participate voluntarily. Participants can withdraw from the study whenever they choose for any reason.

### Eligibility criteria {10}

The following are the criteria for the inclusion of candidates: (1) ≥ 18 years of age; (2) patients and their families fully understand the research and must sign an informed consent form; (3) diagnosed with type 1 or 2 diabetes mellitus; (4) hemoglobin A1c (HbA1c) levels of < 10% within 3 months; (5) moderate or severe nonproliferative diabetic retinopathy (NPDR) and proliferative diabetic retinopathy (PDR) without vitreous/preretinal hemorrhage [33]; (6) clear media for adequate OCT and OCTA images; (7) treatment-naïve DME diagnosed clinically; (8) CST of > 300 μm; (9) Early Treatment Diabetic Retinopathy Study (ETDRS) BCVA between 24 and 73 letters on the day of randomization; (10) treatment within 12 months of DME diagnosis; (11) no contraindication of vitrectomy or conbercept intravitreal injection. Each participant will only have one eye examined. If both eyes are qualified, the researcher will decide which of the eyes will be included in this study.

Exclusion criteria include the following: (1) any previous DME treatment (i.e., anti-VEGF injections, intravitreal corticosteroid usage, or macular photocoagulation); (2) macular edema caused by other diseases (i.e., age-related macular degeneration, retinal vein occlusion); (3) any previous intraocular surgeries (cataract surgery performed at least three months before study entry will not be exclusionary); (4) vision loss caused by other ocular diseases (i.e., glaucoma, high myopia); (5) a follow-up duration of < 12 months; (6) severe dysfunction of the heart, liver, kidney, lung, or other organs.

### Who will take informed consent? {26a}

Prior to involvement in this study, the principal investigator and delegated sub-investigators will provide potential participants with detailed information about the purpose, procedures, risks, and benefits of the research. Participants will have the opportunity to ask questions and clarify any concerns they may have. Only after receiving this information and providing voluntary consent will individuals be included in the study.

### Additional consent provisions for collection and use of participant data and biological specimens {26b}

Not applicable. There will be no additional consent provisions, and this study will not involve biological specimens.

## Interventions

### Intervention description {11a}

#### Control group

Patients will undergo three intravitreal injections of 0.5 mg conbercept (Chengdu Kanghong Biotech Co., Chengdu, China) per month with a 30-guage syringe needle 3.5–4 mm posterior to the corneal limbus under topical anesthesia [46].

#### Study group

Standard 25-guage PPV will be performed under retrobulbar anesthesia using Constellation vitrectomy system (Alcon. Laboratories, Inc., Fort Worth, TX). Phacoemulsification and intraocular lens implantation will be performed for cataract affecting vision. After the central vitreous is removed, the surgeon will make a complete posterior vitreous detachment to clear the attached posterior hyaloid. The ILM stained with indocyanine green will be peeled up to the vascular arcades. Pan-retinal photocoagulation (PRP) can be performed during surgery if needed. In the end, the vitreous cavity will be filled with balanced salt solution. An experienced surgeon will complete all the surgical procedures.

### Further intervention

Thereafter, each participant will receive pro re nata (PRN) conbercept therapy, with regular monitoring for 12 months, at least 1 month apart for the vitrectomy of the study group and 3 months apart for the first intravitreal injection of the control group. PRN conbercept treatment will be performed if the following criteria are met: (1) existence of recent or persisting cystoid retinal lesions, (2) a decrease of no less than 5 ETDRS letters in BCVA, and (3) an increase of 50 μm or more in CST compared with the best value previously achieved. If high-risk PDR without definite vitreous or preretinal hemorrhage or severe NPDR with high-risk characteristics and reduced visual acuity are present, PRP treatment will be administered. Cataract surgery will also be allowed for cataract that affect vision. PPV will be performed if there exist un-clearing vitreous hemorrhage (1–6 months) and/or tractional retinal detachment. Patients with postoperative ocular hypertension will first receive drug treatment to lower their intraocular pressure. If medication is unable to control persistent high intraocular pressure, they will undergo anti-glaucoma surgery. Other further interventions will be conducted based on the specific circumstances.

### Criteria for discontinuing or modifying allocated interventions {11b}

If serious adverse events occur or are likely to occur that severely impact vision (such as endophthalmitis, retinal damage, etc.), or if patients are unable to tolerate the intervention due to their physical condition, the intervention will be terminated or modified. The patients will withdraw from the study and receive appropriate treatment measures.

### Strategies to improve adherence to interventions {11c}

Participants will receive comprehensive education and counseling sessions, which will help them understand the significance of adhering to the prescribed interventions and address any concerns or misconceptions they may have. Investigators will send regular reminders and notifications to participants to ensure they stay on track with the interventions.

### Relevant concomitant care permitted or prohibited during the trial {11d}

Treatment other than “Further intervention” will be prohibited, while treatment for the fellow eye and management of systemic diseases will be permitted.

### Provisions for post-trial care {30}

All follow-up visits will be conducted in the outpatient clinic. After participants withdraw from the study, they will receive standard clinical care. Compensation for any harm caused by the study will be provided by Tianjin Medical University Eye Hospital according to the regulations.

### Explanation for the choice of comparators {6b}

The changes in CST and BCVA are the primary outcomes in this study. BCVA reflects the visual function of patients under the best-corrected refractive state, and it is widely used to evaluate the efficacy of clinical therapy. It is also a primary goal for patients seeking treatment. CST is an anatomical outcome that can reflect macular edema among patients.

### Outcomes {12}

#### Primary outcome

The primary outcome is the changes in BCVA and CST from baseline to month 12 between the two groups.

#### Secondary outcomes

The secondary outcomes include the changes in BCVA and CST from baseline to months 1, 3, and 6; assessment of SRF, location and size of cystoid change, continuity of the IS-OS layer, and presence and quantity of HRF in OCT at months 1, 3, 6, and 12; assessment of FAZ, superficial capillary density (sVD), and deep capillary density (dVD) in OCTA at months 3, 6, and 12; re-treatment and diabetic retinopathy (DR) development from baseline to month 12; cost-effectiveness analysis at month 12; and vision-related quality of life (VRQL) at months 6 and 12.

### Safety evaluation

Safety evaluation of the study includes any consequences associated with the interventions and course of the disease itself, including ocular hypertension, progression of cataract, corneal abrasion, retinal injury, hyphemia, uveitis or inflammatory reaction, and endophthalmitis.

### Participant timeline {13}

Table 1 outlines the timeline for participant enrollment, interventions, assessments, and visits.

**Table 1:** Schedule

	**Study period**										
	**Enrollment**	**Allocation**	**Post-allocation**								**Close-out**
**Time point**	***T***_**1**_	**0**	***T***_**0**_	T^*^_1(1d)_	T^*^_2(1w)_	***T***_**3(1 m)**_	T^**^_4(2 m)_	***T***_**5(3 m)**_	***T***_**6(6 m)**_	***T***_**7(12 m)**_	***T***_**x**_
Enrollment:											
Eligibility screen	X										
Informed consent	X										
Allocation		X									
Interventions:											
PPV			X								
IVC			X			X	X				
Assessments:											
Demographics data	X										
Medical history	X										
Laboratory examination	X										
Eye examination	X			X	X	X	X	X	X	X	
BCVA	X					X		X	X	X	
IOP	X			X	X	X	X	X	X	X	
OCT	X					X		X	X	X	
OCTA	X							X	X	X	
Direct medical cost										X	
NEI-VFQ-25	X								X	X	
Adverse events			X	X	X	X	X	X	X	X	

*PPV*, pars plana vitrectomy; *IVC*, conbercept intravitreal injection; *BCVA*, best corrected visual acuity; *IOP*, intraocular pressure; *OCT*, optical coherence tomography; *OCTA*, OCT angiography; *d*, day; *w*, week; *m*, month

^*^Only study group

^**^Only control group

### Sample size {14}

The sample size is estimated by the change of BCVA using PASS 15.0 (PASS 15.0.5 NCSS, LLC, USA). According to a published paper [9], mean BCVA change of IVC from baseline to month 12 was 8.2 ± 9.5 ETDRS letters. The maximum difference of 5 ETDRS letters was chosen for non-inferiority margin between groups. The sample size was calculated based on α level of 0.05 and *β* level of 0.2, N1 = N2 = 46. Then, the ultimate sample size is 102 (51 of each group) assuming a 10% dropout rate.

The non-inferiority margin was chosen because 5 ETDRS letters is considered clinical insignificant in practice. And the sample size is also sufficient for analysis of the change of CST based on previous studies [9, 36] (364 ± 88.6 μm and 200 ± 210 μm for IVC and vitrectomy combined with ILM peeling respectively).

### Recruitment {15}

We will promote the study through various channels, including outpatient clinics, social media, and internal hospital announcements, to reach a wide audience. We will regularly assess the progress of participant recruitment and make necessary adjustments to the recruitment strategies to ensure the smooth conduct of the study.

### Assignment of interventions: allocation

#### Sequence generation {16a}

One hundred and two random numbers will be generated by computer-generated algorithm (SPSS software), and the corresponding participants will be evenly distributed to the study group (the first 51 numbers) and the control group (the last 51 numbers) according to the random number sequence.

#### Concealment mechanism {16b}

The allocation sequence will be concealed for both investigators and participants by opaque envelopes. Once patients are enrolled and have signed the informed consent form, they will receive the corresponding intervention based on the group assigned in the envelope.

#### Implementation {16c}

The allocation sequence will be generated by a designated investigator, and the assignment of participants to interventions will be carried out by the same person. Participant enrollment will be conducted by the principal investigator.

### Assignment of interventions: blinding

#### Who will be blinded {17a}

The researchers conducting the assessments will be masked when obtaining and analyzing ophthalmic examination data, which includes BCVA, IOP, OCT, and OCTA images. Data analysts will also be masked to ensure precise statistical results.

#### Procedure for unblinding if needed {17b}

This is an open-label trial, with no blinding for participants and surgeons.

### Data collection and management

#### Plans for assessment and collection of outcomes {18a}

Investigators will timely record the postoperative data after every visit. Two delegated investigators will be responsible for the data monitoring and will check the accuracy of the data every quarter. In cases where two assessors disagreed, a senior physician will be consulted for an additional evaluation.

### BCVA

After refractive error correction and optometric evaluation, BCVA will be tested using ETDRS chart (Precision Vision, Villa Prak, Illinois, USA). The logMAR values for finger counting, hand movement, light perception, and no light perception are 1.85, 2.3, 2.6, and 2.9, respectively [47]. An increase of ≥ 15 ETDRS letters, a change of < 15 ETDRS letters, and a decrease of ≥ 15 ETDRS letters is defined as “improving,” “invariant,” and “worsening” of visual acuity, respectively.

### Intraocular pressure

A non-contact computerized tonometer (CT-1, Topcon, Japan) will be used to measure the intraocular pressure (IOP). The average value obtained from three successive measurements will be noted. An intraocular pressure of at least 25 mmHg or a rise of at least 10 mmHg from the baseline is considered to be ocular hypertension.

### Slit lamp bio-microscopy

The eyes will be thoroughly examined with a 90D indirect ophthalmoscopy lens under a slit lamp.

### OCT

Three-dimensional spectral domain OCT (DRI-OCT Triton, Topcon, Inc, Tokyo, Japan) will be used to acquire all OCT scans. Scanning was centered on the macular fovea with a scan length of 6 mm. The resolution was 5 μm, the scan depth was 4 mm, and the scan mode was horizontal linear scanning of 512 × 128. CST refers to the average thickness of the distance between the retinal pigment epithelium (RPE) and the ILM at the highest point of the retina, which is measured at a circular area surrounding the foveal center with a diameter of 1 mm. Additionally, we will assess SRF, cystoid size (< 100 μm, 100–200 μm, > 200 μm) and location (outer nuclear layer (ONL) or inner nuclear layer (INL)), IS-OS continuity (completely continuous, partly disrupted, or completely disrupted), and presence and quantity of HRF (< 10, 11–20, > 20), vitreomacular interface (detached, vitreomacular adhesion, vitreomacular traction, and epiretinal membrane). The technicians will ensure that the images are centered and of sufficient quality. OCT images will be analyzed and compared preoperatively and at months 1, 3, 6, and 12.

### OCTA

OCTA images will be obtained following a standard protocol with the AngioVue OCTA system (RTVue-XR Avanti, Optovue, Fremont, California, USA). The scanning area, centered on the fovea, was captured in 3 × 3 mm sections with a resolution of 304 × 304 pixels. The in-built RTVue XR Avanti AngioVue software will help reveal FAZ, sVD, and dVD through the automated layer segmentation. Motion artifacts, a weak signal (5/10), poorly focused scans, or segmentation failure will all result in poor image quality, which will be disregarded. OCTA images will be analyzed and compared preoperatively and at months 3, 6, and 12.

### DR classification

DR will be graded and classified according to International Clinical Diabetic Retinopathy and Diabetic Macular Edema Disease Severity Scales [33].

### Cost-effectiveness analysis

Clinical efficacy will be assessed based on BCVA and CST at the last follow-up and compared to that before operation. Cured: BCVA increased ≥ 15 ETDRS letters, CST decreased ≥ 20%, and no cystoid change is observed; improved: BCVA increased ≥ 5 ETDRS letters, CST decreased ≥ 10%, and no cystoid change is observed; invalid: BCVA and CST have no obvious change or deterioration. Effective rate = [*n* (cured) + *n* (improved)]/*n* (total). Costs include direct, indirect, and hidden costs. The registration fees and related examination fees are considered the same, and indirect or hidden costs such as transportation fees and late work fees are ignored. The treatment and drug costs are included in the direct medical cost, which is calculated according to the treatment and drug charge standard in 2023 of Tianjin Third-Level First-Class Hospital. The incremental cost-effectiveness ratio (ICER) is used as the evaluation index in cost-effectiveness analysis. Cost-effectiveness ratio = treatment cost/effective rate. ICER is the ratio of the difference between the treatment costs of the two groups and the difference between the effective rate of the two groups, indicating the cost of each additional effective patient in the study group versus the control group. The average cost of the two groups of treatments is taken as the willingness to pay. If ICER is less than the willingness to pay, the treatment scheme is cost-effective.

### VRQL

A translated, Chinese version of the National Eye Institute Visual Function Questionnaire-25 (NEI-VFQ-25) will be administered preoperatively, as well as at months 6 and 12.

### Plans to promote participant retention and complete follow-up {18b}

Investigators will give participants a detailed account of the follow-up timepoints and emphasize the importance of regular participation. Besides, investigators will supplement with frequent reminders and flexible scheduling options.

### Data management {19}

Electronic Data Capture (EDC) system will be used for data entry. Only authorized researchers will have access to and be able to process the data. Encryption technology will be used to protect data during transmission and storage. The data will be stored on secure servers and databases, which will be subject to strict access control and monitoring to ensure data confidentiality and integrity.

### Confidentiality {27}

All data collected will be coded, and patient identifiers will be removed to maintain anonymity. Electronic data will be stored in a secure, password-protected database accessible only to members of the research team. Paper documents will be kept in a locked cabinet to prevent unauthorized access.

### Plans for collection, laboratory evaluation, and storage of biological specimens for genetic or molecular analysis in this trial/future use {33}

Not applicable. There are no biological specimens.

### Statistical methods

#### Statistical methods for primary and secondary outcomes {20a}

Descriptive statistics will be used to summarize study results and demographic variables. Means and standard deviations will be used to describe the normally distributed data, and medians and interquartile ranges will be used for non-normally distributed data. Categorical variables will be described as percentage and frequency. The two-sample *t*-test or Kruskal–Wallis test will be used to assess continuous variables, while the chi-square test will be used to evaluate categorical variables. A two-sided *p* value of less than 0.05 will be considered statistical significance. Besides, the records of patients with missing data will be deleted to ensure the accuracy of the results. All the data will be analyzed by SPSS 25.0 (Chicago, IL, USA).

### Interim analyses {21b}

The research committee of Tianjin Medical University Eye Hospital will evaluate the progress and safety of the study in the interim. Final decision regarding the termination of the trial will be made jointly by the principal investigator and the regulatory body overseeing the study. They will base their decision on the results of the interim analyses, safety data, and efficacy data as well as ethical and legal requirements.

### Methods for additional analyses (e.g., subgroup analyses) {20b}

Regression analysis will be used for adjusted analysis to control for confounding factors.

### Methods in analysis to handle protocol non-adherence and any statistical methods to handle missing data {20c}

Missing data will be managed using a combination of the last observation carried forward (LOCF) and multiple imputation (MI) methods. Sensitivity analyses will also be performed to assess the impact of different imputation methods on study results.

### Plans to give access to the full protocol, participant-level data, and statistical code {31c}

Access to the full protocol and participant level-data can be acquired on ClinicalTrials.gov.

### Oversight and monitoring

#### Composition of the coordinating center and trial steering committee {5d}

The research committee of Tianjin Medical University Eye Hospital will meet regularly to review the progress of the research, make important decisions, and ensure that the trial adheres to ethical and regulatory standards.

### Composition of the data monitoring committee, its role and reporting structure {21a}

The data monitoring committee comprises two independent statisticians and one independent clinician. They will review the accumulating data every quarter to assess the progress of the study, make important decisions regarding participant safety, and evaluate the integrity of this trial.

### Adverse event reporting and harms {22}

During each follow-up visit, adverse events will be documented and recorded in the EDC system. Severe adverse events will be reported to Tianjin Medical University Eye Hospital Ethics Committee within 24 h of their occurrence.

### Frequency and plans for auditing trial conduct {23}

Regular audits will be conducted every 6 months to monitor and evaluate the conduct of the trial. These audits will be performed by an independent auditing team, consisting of qualified individuals who are not directly involved in the study.

### Plans for communicating important protocol amendments to relevant parties (e.g., trial participants, ethical committees) {25}

Important protocol amendments will be communicated to trial participants through appropriate means, such as written notifications, verbal explanations, or information sessions. Participants will be provided with opportunities to ask questions or express concerns. Important protocol amendments will also be promptly submitted to the ethical committees for review and approval, following their requirements and procedures. Detailed explanations and justifications will be provided to ensure that the amendments receive thorough scrutiny and authorization.

### Dissemination plans {31a}

Study results will be disseminated widely through publications in open-access journals and presentations at conferences both domestically and abroad. The data that support the findings of this study are available from the corresponding author upon reasonable request.

## Discussion

The high incidence of refractory persistent DME necessitates the research for a more efficacious method for treating DME. Based on previous studies [30, 34–36], we have ample reasons to postulate that PPV is safe and effective for treatment-naïve DME. Moreover, PRP and cataract surgery can be performed simultaneously with PPV, obviating the need for continual hospital visits for treatment. However, PPV has mostly been used to treat refractory DME before, and the long-term course of disease and repeated intravitreal injections may have caused irreversible damage to the retina. Hence, we are going to explore whether early PPV combined with ILM peeling can reduce the therapeutic burden of DME patients, prevent vision loss, and maintain long-term stabilization of diabetic retinopathy, thus becoming as an initial therapy choice for treatment-naïve DME patients. Furthermore, with the assistance of OCT and OCTA, we will assess the imaging biomarkers connected with surgical treatment effectiveness.

### Trial status

The study has not yet started recruitment. The protocol is version 2.0, 20231004. The study will last about 2 years, from November 1, 2023, to November 1, 2025.

## Data Availability

Access to the final trial dataset will only be granted to the investigators involved in the study.

## Abbreviations

BCVA: Best corrected visual acuity
CST: Central subfield thickness
DME: Diabetic macular edema
DR: Diabetic retinopathy
DRCR: Diabetic Retinopathy Clinical Research
dVD: Deep capillary density
EDC: Electronic Data Capture
FAZ: Foveal avascular zone
HRF: Hyperreflective foci
ICER: Incremental cost-effectiveness ratio
ILM: Internal limiting membrane
INL: Inner nuclear layer
IOP: Intraocular pressure
IS-OS: Inner segment-outer segment
IVC: Conbercept intravitreal injection
LOCF: Last observation carried forward
MI: Multiple imputation
NEI-VFQ-25: National Eye Institute Visual Function Questionnaire-25
NPDR: Nonproliferative diabetic retinopathy
OCT: Optical coherence tomography
OCTA: Optical coherence tomography angiography
ONL: Outer nuclear layer
PDR: Proliferative diabetic retinopathy
PPV: Pars plana vitrectomy
PRN: Pro re nata
PRP: Pan-retinal photocoagulation
RCT: Randomized controlled clinical trial
RPE: Retinal pigment epithelium
SRF: Subretinal fluid
sVD: Superficial capillary density
VEGF: Vascular endothelial growth factor
VRQL: Vision-related quality of life
